# The effects of cognitive behavioral therapy in women with polycystic ovary syndrome: A meta-analysis

**DOI:** 10.3389/fpsyg.2022.796594

**Published:** 2022-10-26

**Authors:** Rong Tang, Junlan Yang, Yanmei Yu, Yuying Fang

**Affiliations:** ^1^Center for Reproductive Medicine, Cheeloo College of Medicine, Shandong University, Jinan, China; ^2^Shandong Provincial Hospital, Jinan, China; ^3^School of Medicine, Shandong University, Jinan, China

**Keywords:** cognitive behavioral therapy, polycystic ovary syndrome, anxiety, depression, quality of life

## Abstract

**Background:**

Cognitive behavioral therapy (CBT) has well-characterized benefits in alleviating diseases associated with depression, anxiety, and obesity, resulting in a marked improvement in the patient’s quality of life. There are some studies regarding the effects of CBT on patients with polycystic ovary syndrome (PCOS). However, there is still no report of a meta-analysis for systematic assessment.

**Objectives:**

This study aimed to evaluate the effectiveness of CBT in improving weight loss, anxiety, depression, life quality, compliance, and pregnancy outcomes in patients with PCOS.

**Methods:**

Studies regarding CBT related to PCOS in PubMed, Cochrane library, Embase, ClinicalTrials.gov, CNKI, and WANFANG DATA were searched for up to 19 November 2020. A random-effects model was used to perform a meta-analysis.

**Results:**

Eight trials regarding CBT compared with lifestyle modification and routine treatments were included. No differences in depression (SMD –1.11; 95% CI –2.28, 0.07; *P* > 0.05), body mass index (BMI) (SMD 0.88; 95% CI –0.94, 2.71; *P* > 0.05), or overall life quality (SMD 1.24; 95% CI –0.44, 2.92; *P* > 0.05) were evident between CBT and control groups; however, anxiety (SMD –1.12; 95% CI –2.1, –0.13; *P* < 0.05) and quality of life in hirsutism (SMD 0.92; 95% CI 0.48, 1.35; *P* < 0.05) were significantly improved. For secondary outcomes, both patient compliance and pregnancy rate were improved, but no significant change in pregnancy loss rate was identified.

**Conclusion:**

CBT exhibited obvious advantages in the alleviation of anxiety, improvement of quality of life in hirsutism, and increase of compliance and pregnancy rate in patients with PCOS. Larger and higher-quality randomized controlled trials are needed to clarify the role of CBT in PCOS.

**Systematic review registration:**

[https://www.crd.york.ac.uk/PROSPERO/], identifier [CRD42021225856].

## Introduction

Polycystic ovarian syndrome (PCOS) is a common endocrine-metabolic disease, affecting up to 6–12% of women of reproductive age (March et al., 2010; Bozdag et al., 2016). The clinical features of PCOS include hyperandrogenism, abnormal anovulation, polycystic ovary morphology (as revealed by ultrasound), and insulin resistance among others. PCOS usually manifests as a multisystem disorder. Psychological comorbidities are very common in PCOS patients, especially in those suffering from obesity and/or infertility, which may worsen the patient’s condition. A study based on data from the Australian Longitudinal Study on Women’s Health (ALSWH) demonstrated that compared with controls, women with PCOS exhibited an increased risk of depression and anxiety (Damone et al., 2019). Symptoms of PCOS, including reproductive dysfunction, metabolic dysregulation, and poor mental health are cited as reasons for reduced quality of life and increased financial issues (Greenwood et al., 2018; Sanchez-Ferrer et al., 2020). International evidence-based guidelines for PCOS recommend depression and anxiety screening for all women with PCOS and lifestyle modification as the first line of treatment (Teede et al., 2018). To improve patient’s quality of life, the management of depression and anxiety is essential.

Cognitive behavioral therapy (CBT) is a type of psychotherapy that modifies cognitive patterns and provides behavioral restructuring to alter psychological symptoms and behaviors. CBT has been successful in the treatment of various illnesses including obesity, depression, anxiety, eating disorders, insomnia, and other chronic disorders (Grilo et al., 2011; Reavell et al., 2018; Zweerde et al., 2019). CBT is recommended by the American Psychological Association and the American College of Physicians as the first-line treatment for depression (Qaseem et al., 2016). International evidence-based guidelines for PCOS also recommend CBT to help patients adhere to and maintain healthy lifestyles and improve health outcomes (Teede et al., 2018). There have been several studies on the effects of CBT on depression, anxiety, quality of life, pregnancy, pregnancy loss, and compliance in women with PCOS. However, to our knowledge, no meta-analysis has been performed regarding these analyses. The primary aim of this study was to conduct a meta-analysis to explore the effects of CBT on depression, anxiety, and quality of life in women with PCOS.

## Materials and methods

### Search strategy

Two of the authors searched PubMed, Cochrane library, Embase, ClinicalTrials.gov, CNKI, and WANFANG DATA for studies published up until 19 November 2020. The search terms can be found in Supplementary Appendix. The review was prospectively registered on PROSPERO (registration number CRD42021225856).

### Selection criteria

Studies were included in the present meta-analysis if (1) women were diagnosed with PCOS by Rotterdam criteria, NIH criteria, ESHRE/ASRM criteria, or China clinical guidelines; (2) the types of the trial included were randomized controlled trials (RCTs) and cohort studies; (3) only studies that included at least one treatment condition described as CBT intervention or as being based on CBT principles were eligible, where patients were taught cognitive skills such as identifying automatic thoughts and cognitive distortions; (4) studies in which control groups underwent merely lifestyle modification or routine treatments (medications and standard care); and (5) papers provided sufficient data to calculate effect sizes regarding changes in weight, depression, anxiety, and/or quality of life. Studies were excluded if (1) a full text was unavailable and (2) studies were overlapping.

### Data extraction

Two reviewers independently assessed the eligibility of the identified studies and extracted the data based on the inclusion criteria. Any disagreements regarding inclusion were resolved by consensus or arbitration. Information regarding general characteristics of the study (author, age, year of publication, country region), diagnostic criteria for PCOS, primary outcomes (weight, depression, anxiety, and quality of life scores), secondary outcomes (compliance rates, pregnancy rates, and abortion rates), sample size, and intervention were gathered.

### Quality assessment

Two authors of the present review independently assessed the risk of bias in these included studies using Cochrane Collaboration’s risk of bias tool for RCTs and the Newcastle-Ottawa Scale for cohort studies. Any disagreement or uncertainty was resolved by discussion among reviewers until a consensus was reached.

### Data analysis

For continuous data, the results were pooled and an SMD was calculated. For dichotomous data, risk ratios (RRs) were calculated among the control and intervention groups. Heterogeneity between the studies was assessed using I^2^ statistical analysis (I^2^ statistic > 70% was allocated as high heterogeneity) and 95% confidence intervals (CIs) were calculated for all outcomes. A random effects model was used for further data analysis. STATA 16 was used for direct meta-analysis.

## Results

### Characteristics and quality of the included studies

**FIGURE 1:**
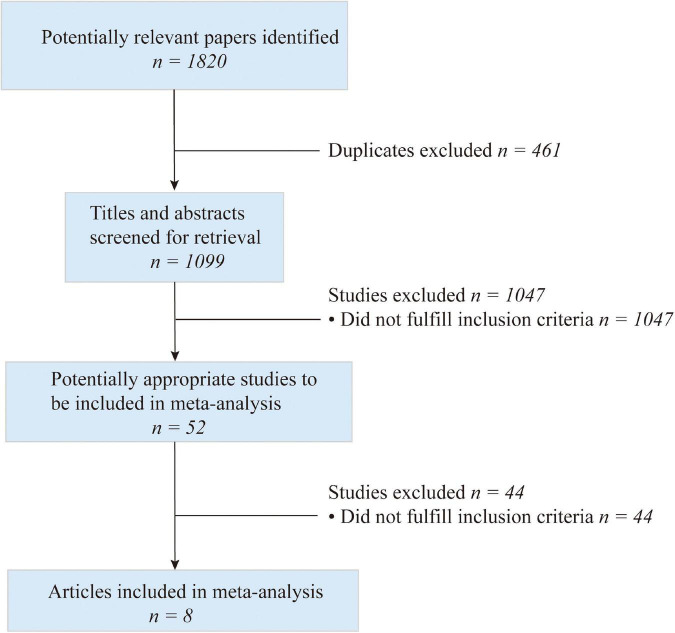
Flow chart of study selection.

The initial search yielded 1,560 articles. A total of 1,099 articles were screened and 461 duplicate articles were excluded. By reading titles and abstracts, 1,047 articles were excluded and 52 articles were further assessed in full-text form. After the assessment, 44 articles were excluded by conforming to the inclusion/exclusion criteria. Finally, eight trials that matched all criteria were analyzed in this meta-analysis (Figure 1). The characteristics of the studies are presented in Table 1. Information extracted included author, age, geographical country/region, sample size, intervention, and outcomes. The details of quality assessment within individual studies are presented in Table 2. Of the eight included studies, there were some concerns for two RCTs and the other ones had a low risk of bias. For cohorts, the qualities of the four studies were poor.

**TABLE 1:** Characteristics of the included studies.

Study	Country region	Diagnostic criteria	Mean age		Sample		Intervention		Outcomes	Scales	Measurement timepoint	Duration of CBT^a^	Constituents
			Experiment group	Control group	Experiment group	Control group	Experiment group	Control group					
Cooney et al. (2018)	USA	NIH	29 ± 5.9	32 ± 5.2	7	8	CBT + LS^b^	Only LS	BMI depression anxiety quality of life	CES-D^f^ STAI^g^ PCOSQ^h^	16 w	Weekly 30-min for 16 w	Group intervention
Abdollahi et al. (2018)	Iranian	Rotterdam Criteria	28 ± 4.4	27 ± 4.6	37	37	Received CBT based on routine treatments^c^	Only routine treatments	BMI depression quality of life	BDI^i^ PCOSQ	4 w^d^	Weekly 45–60 min for 8 w	Group intervention
Liang (2016)	China	Unclear	24.4 ± 4.6	24.5 ± 4.3	29	29	Received CBT based on routine treatments	Only routine treatments	Compliance quality of life	GQOLI-74^j^	Unclear	Unclear	Group intervention
Wang (2019)	China	Chinese Criteria	29.82 ± 2.33	30.15 ± 2.18	50	47	CBT + LS	Only routine treatments	Compliance	_	Unclear	1.5–2 h/time	Group intervention
Sun (2020)	China	Unclear	31.26 ± 4.05	31.64 ± 3.71	43	43	CBT + LS	Only routine treatments	Compliance pregnancy rates	_	12 m^e^	Unclear	Group intervention
Tang (2020)	China	Unclear	27.14 ± 3.75	26.97 ± 3.88	35	35	Received CBT based on routine treatments	Only routine treatments	Depression anxiety pregnancy rates abortion rates	SAS^k^ SDS^l^	3 m	Unclear	Group intervention
Xiao and Yang (2020)	China	Unclear	35.27 ± 1.99	32.16 ± 2.66	37	37	Received CBT based on routine treatments	Only routine treatments	anxiety pregnancy rates abortion rates	STAI	Unclear	Unclear	Group intervention
Lin et al. (2018)	China	Unclear	26.2 ± 7.5	27.3 ± 9.6	49	35	Received CBT based on routine treatments	Only routine treatments	Depression anxiety pregnancy rates abortion rates	STAI BDI FertiQol^m^	Unclear	1.5–2 h/time	Group intervention

a, cognitive behavioral therapy; b, lifestyle modification, including diets and exercise; c, received medicines, such as metformin; d, weeks; e, months; f, Center for Epidemiologic Studies Depression Scale; g, State-Trait Anxiety Inventory; h, Polycystic Ovary Syndrome Health-Related Quality of Life Questionnaire; i, Beck depression questionnaires; j, Generic Quality of Life Inventory-74; k, Self-Rating Anxiety Scale; l, Self-Rating Depression Scale; m, fertility quality of life questionnaire.

**TABLE 2:** Summary of risk of bias assessment.

(A)									

Study	Randomization	Concealed allocation	Blind (participants)	Blind (measurers)	Dropout rate	Incomplete outcome data	Selective reporting	Other bias	Risk of bias
Cooney et al. (2018)	Unclear	Unclear	Unclear	Unclear	High	Low	Low	Unclear	Some concerns
Abdollahi et al. (2018)	Unclear	Low	Unclear	Unclear	Low	Low	Unclear	Unclear	Some concerns
Liang (2016)	Unclear	Unclear	Unclear	Unclear	Unclear	Unclear	Unclear	Unclear	High
Xiao and Yang (2020)	Low	Unclear	Unclear	Unclear	Unclear	Unclear	Unclear	Unclear	High

**(B)**

**Study**	**Selection**				**Comparability**	**Outcome**			**Total**
	**Represent of the exposed cohort**	**Selection of the non-exposed cohort**	**Ascertainment of exposure**	**Demonstration that outcome of interest was not present at start of study**	**Comparability of cohorts on the basis of the design or analysis**	**Assessment of outcome**	**Was follow-up long enough for outcomes to occur**	**Adequacy of follow up of cohorts**	

Wang (2019)	*	*	*	*	*				5
Sun (2020)	*	*	*	*	*		*		6
Tang (2020)	*	*	*	*	*	*			6
Lin et al. (2018)	*	*	*	*	*	*			6

* means graded 1 score.

### Data synthesis and meta-analysis

#### Primary outcomes

##### Body mass index and psychological

**FIGURE 2:**
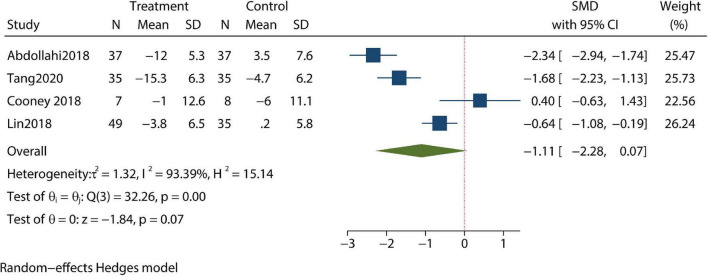
Meta-analysis of depression.

**FIGURE 3:**
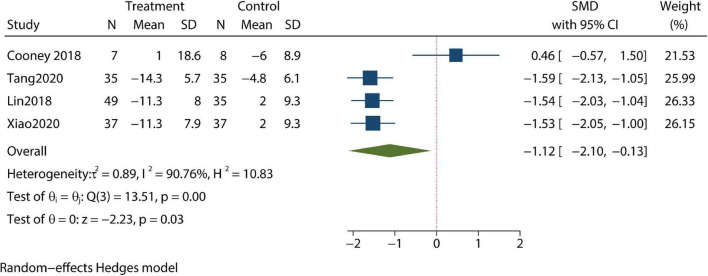
Meta-analysis of anxiety.

**FIGURE 4:**
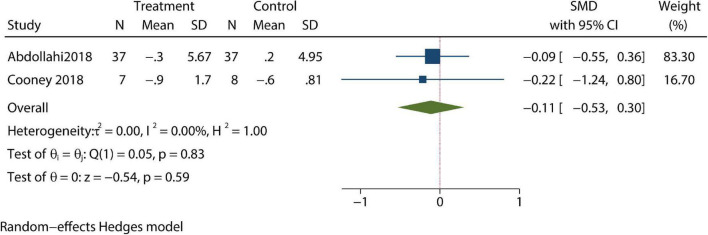
Meta-analysis of BMI.

Changes in depression were reported in four trials; and in these trials, patients’ depressive symptoms were not significantly improved when compared with control groups after review by our meta-analysis (SMD –1.11; 95% CI –2.28, 0.07; *P*>0.05) (Figure 2). Meanwhile, post-treatment anxiety symptoms were studied in another four articles; in these studies, CBT resulted in significant improvements in anxiety symptoms and reduced measured anxiety scores (SMD –1.12; 95% CI –2.1, –0.13; *P* < 0.05) (Figure 3). Changes in body mass index (BMI) were reported in two trials. In the review of the two trials, we failed to demonstrate that CBT had a beneficial impact on BMI (SMD –0.11; 95% CI –0.53, 0.3; *P* > 0.05) (Figure 4).

##### Quality of life

Quality of life is defined as a multifactorial concept that encompasses physical, emotional, and social aspects. Quality of life including menstrual, hirsutism, weight, infertility, emotional, and social aspects were assessed in this study. CBT was identified as improving patients’ quality of life in aspects of hirsutism by our meta-analysis. And no effect was identified when analyzing differences in quality of life in menstrual, infertility, social, weight, and emotional aspects between CBT-treated patients and controls (Table 3).

**TABLE 3:** Meta-analysis of quality of life.

Outcomes	Studies (N)	SMD	95% CI	*P*	I2 (%)
Quality of life	2	1.24	–0.44, 2.92	0.15	87.87
Menstrual	2	0.33	–1.33, 1.99	0.69	87.97
Infertility	2	0.74	–0.97, 2.46	0.39	88.91
Hirsutism	2	0.92	0.48, 1.35	0	0
Weight	2	0.88	–0.94, 2.71	0.34	90.13
Emotion	3	0.96	–0.05, 1.97	0.06	87.01
Social	2	0.44	–0.48, 1.37	0.35	85.96

##### Secondary outcomes

**FIGURE 5:**
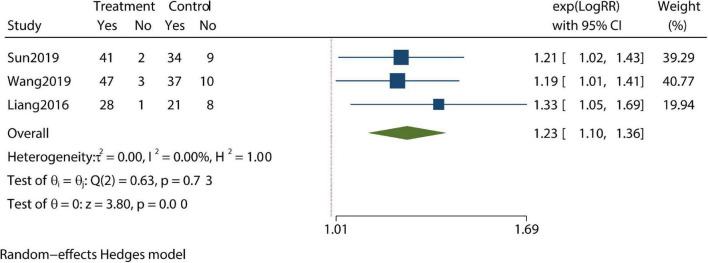
Meta-analysis of compliance rate.

**FIGURE 6:**
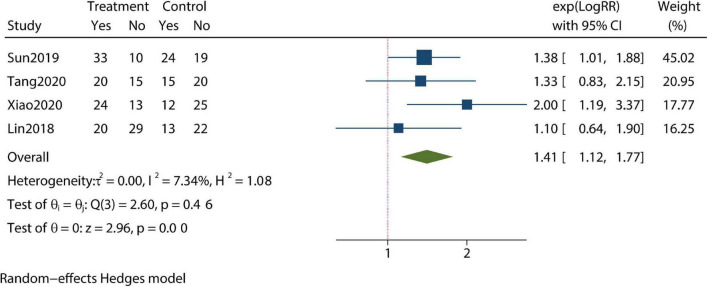
Meta-analysis of pregnancy rate.

**FIGURE 7:**
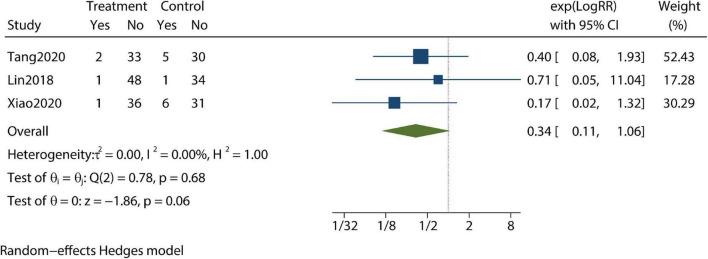
Meta-analysis of pregnancy loss rate.

Patients’ compliance level and clinical pregnancy rate were identified as being improved compared with controls in our meta-analysis (Figures 5, 6). Furthermore, the pregnancy loss rate was reported in three articles and the early pregnancy loss rate was not significantly different between CBT intervention groups and control groups (Figure 7).

## Discussion

To our knowledge, no meta-analysis regarding whether PCOS patients may benefit from CBT was previously available. The present data revealed that CBT has positive effects on anxiety and quality of life in hirsutism aspects in PCOS. Furthermore, our data also suggested that CBT could improve compliance and pregnancy rate. These favorable effects of CBT are outcomes of complex interactions between cognitive, emotional, and behavioral psychological phenomena. For other outcomes, the effectiveness of CBT was not confirmed; these included depression, weight loss, and quality of life in menstrual, infertility, social, weight, and emotional aspects. Moreover, there was insufficient evidence to demonstrate the effectiveness of CBT in reducing the pregnancy loss rate.

Compared with non-PCOS women, women with PCOS are at a higher risk of obesity. Furthermore, obesity is associated with a risk of depression and anxiety (Lindberg et al., 2020; Tyrer et al., 2020). Anxiety, depression, and obesity can interact in those PCOS, resulting in a serious reduction in quality of life. Early intervention, aiming to break the cycle of psychological comorbidities and obesity, exhibit the potential to improve quality of life dramatically and substantially reduce future complications. Psychological comorbidities are a major priority to be addressed in research and clinical domains, with a focus on improving the quality of life of patients with PCOS. Several meta-analyses have revealed that CBT has significant effects on alleviating patient anxiety and related disorders and improving the quality of life of patients with anxiety disorders (Hofmann et al., 2014; Carpenter et al., 2018; James et al., 2020). In line with these studies, our analysis identified that CBT could ameliorate anxiety symptoms in PCOS patients. In those with PCOS, quality of life is increasingly emphasized as an essential patient-reported outcome and can include physical, social, and emotional aspects. Bazarganipour et al. (2015) performed a systematic review focusing on the effects of PCOS on specific aspects of quality of life; it was identified that quality of life was impaired most significantly in hirsutism and menstruation aspects in PCOS. Our study identified solid evidence to the tone that CBT has positive effects on the quality of life in PCOS patients. Great improvements in the hirsutism aspects of quality of life were demonstrated in the present meta-analysis. The effects that CBT has on the quality of life might be constrained mainly to the above aspects, but there is no clear conclusion thus far.

A meta-analysis targeting multiethnic and minority obese adults reported that lifestyle modification based on multi-component programs resulted in reasonable weight loss (Seo and Sa, 2008). However, in our study, we observed no significant effect of CBT on weight loss in patients with PCOS. It is considered that CBT should aid patient participation in lifestyle improvement via support and insistence. DuRant et al. (2009) suggested that the theories of CBT combined with behavioral science could be utilized in a combined manner to overcome the barriers to adapting to a healthy lifestyle. Moreover, we identified one study of non-controlled prospective cohorts that indicated a positive effect when CBT was provided during an intensive lifestyle modification in PCOS with multiple comorbidities such as obesity and depression (Rofey et al., 2009). It was reported that obese individuals with depression achieved weight reduction and symptoms of depression and CVD risk factors were ameliorated (Faulconbridge et al., 2011). However, there was not enough evidence identified in our literature review to support whether effects on weight loss and quality of life could be further optimized by combined CBT and lifestyle interventions in PCOS. Additionally, compliance and dropout in a lifestyle intervention for overweight and obese individuals are noticeable problems that cannot be overlooked. Regarding lifestyle intervention for overweight and obese infertile women, a meta-analysis reported the dropout rate as being up to 24% (Mutsaerts et al., 2013). As expected, the present meta-analysis revealed that compliance levels in PCOS patients could be improved by CBT treatment. CBT is based on three main principles including the teaching of skills, perception alteration, and cognitive reframing to aid patients and improve their ability to control psychological aspects of their disease and induce positive adaptive changes for the effective self-management of symptoms (Dryden, 2005). The economic value of the CBT depends on how its improvements on depression, anxiety, quality of life, compliance, and dropout confer additional health benefits. As PCOS is a chronic disease that requires lifestyle modification, CBT is an ideal therapy to be utilized to control disease effectively and reduce treatment dropout rate. The fundamental proposition of CBT is that cognitive activity, in addition to lifestyle modification or psychological counseling, affects behavior. It has been broadly accepted that desired behavioral changes may come about through cognitive change. As a routine psychosocial care, CBT provided by fertility staff or clinical psychologists could provide PCOS patients with information about lifestyle and behavioral changes that may negatively affect their general and reproductive health. Fertility staff or clinical psychologists should provide preparatory information regarding medical procedures that should promote compliance with medical treatments, as well as support patients in changing lifestyle behaviors.

This meta-analysis has numerous strengths. Several databases were searched without restrictions on language or timescales. One of the strengths of our study is that three common complications including obesity, depression, and anxiety in patients with PCOS have been studied simultaneously. Additionally, quality of life and pregnancy outcomes were also evaluated. However, there are also several limitations in the present study. The first is the small number of literature because studies that specifically address cognitive behavioral therapy for PCOS are few. The second is that several studies have low quality. Some studies lack a specific description of the dose of CBT and duration of treatment, and some studies lasted less than 6 months, while 6 months is usually necessary for a patient to exhibit behavioral change (Abdollahi et al., 2018; Cooney et al., 2018; Tang, 2020). CBT includes three-component intervention (nutrition, exercise, and CBT), which needs a professional counselor. The time-intensive nature of the implementation of CBT was also noticeable. It is possible that the brief descriptions of treatments provided may not fully reflect how the treatment is being delivered in practice. Finally, heterogeneity is an issue that cannot be ignored. There was substantial heterogeneity between study participants, outcome scales, CBT content, and delivery, which may influence the findings. Given the limited studies and sample size, we cannot conduct sub-analysis and sensitive analysis to explore the origin of the heterogeneity. In addition, a questionnaire of self-reported outcomes could result in bias in several studies.

In future, more well-designed clinical trials are needed to explore the effects of CBT when combined with lifestyle intervention in PCOS patients. Meanwhile, long follow-ups are required to observe the long-term effects of CBT in patients with PCOS.

## Conclusion

Women with PCOS are at higher risk of depression and anxiety and have a significantly lower quality of life compared with non-PCOS individuals. The meta-analysis presented herein reveals that CBT has obvious advantages in alleviating PCOS patients’ anxiety symptoms and improving their quality of life in aspects of hirsutism. Moreover, there is overall evidence indicating that CBT improves patient compliance and pregnancy rates. However, there is no substantial evidence suggesting whether CBT has any effect on patient BMI or quality of life in menstrual, infertility, social, weight, and emotional aspects. Therefore, further randomized, double-blind trials with a large sample size and sufficient follow-up time are required in future research.

## Data availability statement

The original contributions presented in this study are included in the article/Supplementary material, further inquiries can be directed to the corresponding author/s.

## Author contributions

JY and YF: data curation. RT: funding acquisition, investigation, and writing – review, and editing. JY and YY: methodology and writing – original draft. All authors contributed to the article and approved the submitted version.
