# Supplementary material for: The effects of cognitive behavioral therapy in women with polycystic ovary syndrome: A meta-analysis
Source: Front Psychol. 2022 Oct 26;13:796594. doi: 10.3389/fpsyg.2022.796594 (PMC9643337; doi:10.3389/fpsyg.2022.796594)
Supplement: Supplementary file 2 [file Data_Sheet_2.PDF]

#1 Polycystic Ovary Syndrome [MESH]

#2 Polycystic Ovar* [title/abstract]

#3 PCOS OR PCOD [title/abstract]

#4 sclerocystic ovar*[title/abstract]

#5 stein Leventhal [title/abstract]
#6 hirsut*.[title/abstract]
#7 anovulat*.[title/abstract]
#8 or/1 - 7

#9 BEHAVIOR THERAPY [MESH]

#10 Psychotherapy[MESH]

#11 CBT[title/abstract]

#12 cogniti* AND (behavio* OR intervention* OR psychotherapy* OR technique* OR therap* OR treat*)[title/abstract]

#13 behavio* AND (intervention* OR psychotherapy* OR technique* OR therap* OR treat* OR activat* OR modif* OR change*)[title/abstract]

#14 or/9-13

#15 #8 AND #14
